# Children, sealants, and guardians who smoke: Trends in NHANES 2001-2002 to 2010-2012

**DOI:** 10.15761/DOCR.1000102

**Published:** 2015-01-29

**Authors:** R. Constance Wiener

**Affiliations:** Dental Practice and Rural Health, School of Dentistry, Department of Epidemiology, School of Public Health, West Virginia University, USA

**Keywords:** dental sealants, tobacco use, NHANES

## Abstract

**Objective:**

There are many factors influencing dental behavior. The relationship of smokers who smoked inside the home toward preventive care (measured as dental sealant placement) of the children living in their homes is examined in this study.

**Methods:**

Data from the National Health and Nutrition Examination Surveys in 2001-2002 and in 2011-2012 were analyzed. Data included variables to someone smoking inside the home, dental sealant placement in children ages 6-less than 20 years, and sociodemographics which were obtained from a dental examination and a home interview.

**Results:**

There were 3,352 eligible participants in 2001-2002 and 2,374 in 2011-2012. The unadjusted odds ratio for not having dental sealants when there was someone who smoked inside the home as compared with not having dental sealants when there was no one who smoked inside the home was 1.57 (95%CI: 1.17, 2.10) in 2001-2002. The unadjusted odds ratio was 1.56 (95% CI: 1.20, 2.03) in 2011-2012. When the data were adjusted for age, sex, race/ethnicity, insurance, and income to poverty ratio, the 2001-2002 adjusted odds ratio was 1.31 (95%CI: 0.97, 1.78). The adjusted odds ratio in 2011-2012 was 1.41 (95% CI:1.01, 1.95).

**Conclusions:**

Children who lived in homes in which someone smoked inside the home were more likely to not have dental sealants compared with children who lived in homes in which no one smoked inside the home. These results are important for understanding the factors related to access to dental care issues for children.

## Background

In 1979, the Department of Health and Human Services developed a 10-yearpublic health goal for the people of the United States. Since then, 10-year goals and programs were developed in advance of the years 2000 (Healthy People 2000), 2010 (Healthy People 2010) and 2020 (Healthy People 2020). In addition to addressing the biological factors involved with health, the programs also included social, environmental, and behavioral factors. Major objectives in all of the programs were reductions in tobacco use and reductions in secondhand smoke exposure. The U.S. National Cancer Institute identified secondhand smoke as exposure to smoke from burning end of tobacco and/or exposure to the exhaled smoke of the smoker [1]. Tobacco use and secondhand smoke exposure are significant global public health problems. Secondhand smoke is a factor in heart disease, lung cancer, asthma attacks, respiratory and ear infections, sudden unintentional infant death syndrome [2-5] and early childhood caries [6,7]. There are over 7000 components in secondhand smoke and some are more concentrated than in firsthand smoke [1,8,9].

Specific examples of the Healthy People 2020 tobacco objectives include: an increase in tobacco screenings in dental care settings (from 52.9% in 2010 to a proposed 58.2% in 2020); a reduction in the use of cigarettes by adults (from 20.6% in 2008 to a proposed 12% in 2020); an increase in smoking cessation attempts by adults (from 48.3% in 2008 to a proposed 80% in 2020); and a reduction in the proportion of children ages 12-17 years exposed to secondhand tobacco smoke (from 45.5% in 2005-08 to a proposed 41% in 2020) [10]. The Surgeon General's Report on Oral Health in America stressed that cultural/ behavioral factors (such as tobacco use) affect how people care for themselves and seek and use health services [11].

Healthy People 2020 also addresses the provision of quality care (better health care, better preventive care), and having equitable care to reduce health disparities. One of the oral health objectives is to have more children receive dental sealants. Bisphenol-a glycidyl dimethacrylate (BIS-GMA) dental sealants were introduced in the 1970s to protect the occlusal surfaces of teeth from dental caries and they are an underutilized service. The 2020 proposed objective for children ages 6-9 years is to have 28.1% receive molar sealants (up from 25.5% in 1999-2004); and for children ages 13-15 years, the objective is to have 21.9% receive molar sealants (up from 19.9% in 1999-2004) [10].

Social and cultural/behavioral factors of oral health have been implicated in oral health disparities [12]. Income, race/ethnicity, and education are among the most studied social and cultural/behavioral factors. During the decade from 2001 to 2011, many social and cultural/behavioral changes have occurred. Considering income, between 2001 and 2011, many people in the United Sates have had financial hardships resulting from direct and indirect financial impact of the 9/11 Terrorist Act, the wars in Iraq and Afghanistan, the Great Recession, the economy's slow recovery, and job and investment losses. In addition to the events having an impact on income, they also had public health consequences. In dentistry, dental treatment stagnated for children of age 0-20 years. In 2001, dental expenditures for individuals ages 0-20 years were $666 per patient, and in 2011, the dental expenditures were $649 per patient [13]. In terms of education for that time frame, the median U.S. education level in 2000 and 2013 was a high school education with 28.6% having a high school education in both 2000 [14] and 29.5% in 2013 [15].

Tobacco use is a cultural/behavioral factor of oral health which has changed in prevalence from 2001 to 2011. Social pressure to reduce smoking in public places and social awareness campaigns so that children are not exposed to tobacco smoke in the home have made progress in reducing tobacco use. In 2001, 22.8% of the adults in the U.S. smoked [16] and in 2011, 19.0% of adults in the U.S. smoked [17]. Tobacco use has the potential to synergistically influence the health of a child by not only exposing the child to secondhand smoke, but by also limiting monetary resources for nutritious food and healthcare through the expenditure of money on tobacco. A pack-year of cigarettes costs over $2000 [18]. Also, previous research has indicated that tobacco users were more likely to perceive a need for dental treatment in all categories except dental cleaning (prevention) services [19].

The purpose of this research is to:

- Describe the frequency of sealant placement in 2001-2002 and in 2011-2012 for children who live in homes with someone who smokes inside the home;
- Describe the association of sealant placement and someone smoking inside the home with sealant placement in children who do not have someone smoking inside the home;
- Compare the association of sealant placement and someone smoking inside the home in 2001-2002 with 2011-2012.

The rationale for this study is that someone who smokes inside a home may be more likely to not seek dental preventive services for the children in the home given his or her own less perceived need for dental cleaning (preventive services). The potential exists despite the public health efforts of Healthy People 2010. Dye, *et al*. reported that the association of smoking and culture may guide decision-making and rationalizing the need for care/dental utilization [19]. This study furthers that research into the influence on the children in the home of someone who smokes inside the home. That is, the attitudes of someone smoking inside the home toward preventive dental services for the children may not have been influenced by social pressures and social awareness campaigns for the sealant objectives of Healthy People 2010. Previous researchers have not examined the association of tobacco use inside the home and its association with the preventive care of children as a cultural/behavioral factor of oral health care.

The null hypothesis is that the odds ratios for no sealant placement in children from homes with someone smoking inside the home and for children who do not have someone smoking inside the home is 1.00. The research hypothesis is that the odds for no sealant placement is greater than 1.00 for children from homes with someone smoking inside the home as compared with children who do not have someone smoking inside the home.

## Methods

This study was acknowledged by the West Virginia University Institutional Review Board, proposal 1409429938. NHANES, the National Health and Nutrition Examination Survey, is a survey conducted by researchers from the Centers of Disease Control and Prevention (CDC). It includes interviews, examinations and laboratory tests on the health, and nutrition of noninstitutionalized civilians in the United States. It has a complex survey design with oversampling of subgroups to increase the reliability and precision of estimates. The NHANES researchers used a complex, multistage probability design with weighting schemes. All participants were provided with verbal and written consent. Each year of the NHANES had approximately 5,000 participants. Details of the NHANES studies are provided at the NHANES website [20].

The data used in this study are from the public release of NHANES 2001-2002 and NHANES 2011-2012 data.

### Inclusion criteria

The study design for this study was cross-sectional. Participants were selected from the NHANES 2001-2002 and NHANES 2011-2012 data sets. Inclusion criteria were that the participants were between the ages of 6 and 20 years, had oral evaluations including sealant placement data, and had yes/no data concerning the presence or absence of someone smoking in the home.

### Variable of interest, sealant placement

The participating children had oral health examinations conducted by calibrated dentists who held a state dental license. The examinations were conducted in the NHANES mobile examination center. The examiners used a surface reflecting mirror and number 23 explorers. The teeth were air-dried before evaluation. In this study, a sealant was identified as being present when any sealant material was present on the surface of the occlusals of the premolars, primary molars, or first and second molars; however if the sealant appeared to be part of a restoration rather than a preventive service, the tooth was identified as having a restoration rather than a sealant [20].

### Variable of interest, someone smoking in the home

The variable, presence or absence of someone smoking in the home, was determined by the response of one of the family members answering the question about the smoking behavior of all household members. The question was asked in the home as part of the Family Questionnaire. The question was: “Does anyone who lives here smoke cigarettes, cigars, or pipes anywhere inside this home?” [20].

### Other variables

Other variables considered in the study were sex (male v. female); age (6 to less than 12 years v. 12 to less than 20 years); race/ethnicity (Non-Hispanic Black, Mexican-American, Other v. Non-Hispanic White); family income to poverty ratio (1 to less than 1.25, 1.25 to less than 2, 2 to less than 4.00 v. 4.00 and above); and insurance (no v. yes).

### Statistical methods

Data were analyzed for sample characteristics (frequency, weighted percentages, and standard errors), and frequency of sealant placement in all children and in children who lived in households in which someone smoked inside the home. Chi square analyses were conducted for children ages 6 to less than 12 years and 12 to less than 20 years who also lived in household in which someone smoked inside the home versus sealant placement in 2001-2002 and 2011-2012. Unadjusted and adjusted logistic regression on no sealant placement were conducted for children ages 6 to less than 20 years who lived in households in which someone smoked inside the home versus children ages 6 to less than 20 years who lived in household in which no one smoked inside the home. The significance level of 0.05 was used. All statistical analyses were conducted with SAS 9.3 (SAS Institute Inc., Cary, NC).

## Results

### Sample characteristics

The sample in 2001-2002 and 2011-2012 had an equal distribution of male and female children: 48.7% females in 2001-2002 and 48.7% in 2011-2012. There were no significant differences in sample characteristics ofagerace/ethnicity, family income to poverty ratio, or insurance coverage between 2001-2002 and 2011-2012. The samples had 43.9% ages 6 years to 12 years in 2001-2002, and 42.1% in 2011- 2012. There were 59.9% Non-Hispanic whites in 2001-2002, and 54.4% in 2011-12. There were 29.8% (2001-2002) and 33.5% (2011-2012) who had a family to poverty ratio of 0 to less than 1.25. In 2001-2002, 84.5% had insurance coverage and in 2011-2012, 89.6% had insurance coverage. There was a significant increase in overall sealant placement in 2011-12 from 33.1% in 2001-2002 to 41.2% in 2011-12 (*p*=0.0063). There was a significant decrease in someone who smoked inside the home in 2012 from 22.6% in 2001-2002 to 10.9% in 2011-2012 (*p*<0.001) (Table 1).

### Prevalence and Chi-Square analysis

**Figure 1:**
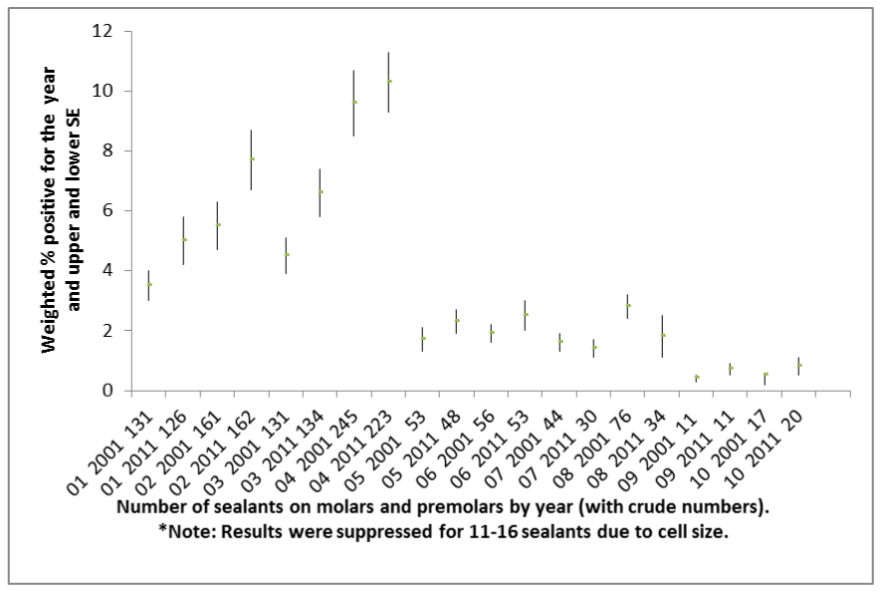
Prevalence of Sealant placement, overall

**Figure 2:**
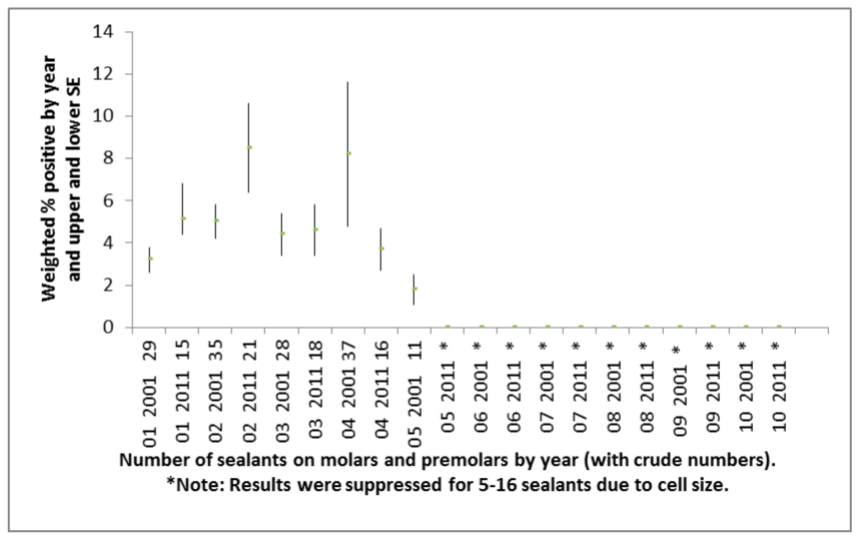
Prevalence of Sealant placement, & smoking inside the home

Prevalence of sealant placement on molars and premolars is presented in Figure 1. In 2001 there were 19.8% participants who had 4 or more sealants; and in 2011, there were 22.0% who had 4 or more sealants. Prevalence of sealant placement on molars and premolars when there was someone who smoked inside the home is presented in Figure 2. In 2001-2002, there were 13.4% of children who had 4 or more sealants and had someone who smoked inside the home and in 2011-2012 there were 13.9% of children who had 4 or more sealants and someone who smoked inside the home.

Chi-Square analyses with respect to household smoking comparing 2001-2002 and 2011-2012 are presented in Tables 2 and 3. From 2001- 2012, there was no significant difference in sealants in children ages 6 to less than 12 years, nor in children 12 to less than 20 years who had someone who smoked inside the home. In subgroup analysis considering sex, race/ethnicity, and family income to poverty ratio, the only significant relationship was for Non-Hispanic Black children ages 6 to less than 12 years in which there was an increase in sealants from 2001-2002 to 2011-2012. That is, in 2001-2002, there were 17.5% of Non-Hispanic black children ages 6 to less than 12 years living in a home in which someone smoked inside the home who had sealants. The weighted percentage was 31.4% in 2011-2012.

Although not presented in tabular form, there were 30.5% (Standard Error, SE, 3.1) of children ages 6 to less than 12 years who lived in homes in which no one smoked inside the home who received sealants in 2001-2002, and there were 40.4% (SE=2.2) who received sealants in 2011-2012. There was a significant increase in sealant placement for the children ages 6 to less than 12 years who lived in homes in which no one smoked inside the home from 2001-2002 to 2011-2012 (p=.0104). For the children ages 12 to less than 20 years who lived in homes in no one smoked inside the home, 39.2% (SE=1.9) received sealants in 2001-2002, and 43.7% (SE=3.1) received sealants in 2011-2012. There was no difference in sealant placement for the children ages 12 to less than 20 years who lived in homes in which no one smoked inside the home from 2001-2002 to 2011-2012 (p=.2117).

### Logistic regressions

The unadjusted odds ratio for not having dental sealants when there was someone who smoked inside the home as compared with not having dental sealants when there was no one who smoked inside the home was 1.57 (95%CI: 1.17, 2.10) in 2001-2002. The unadjusted odds ratio was 1.56 (95% CI: 1.20, 2.03) in 2011-2012.

When the data were adjusted for age, sex, race/ethnicity, insurance, and income to poverty ratio, the 2001-2002 adjusted odds ratio was 1.31 (95%CI: 0.97, 1.78). The adjusted odds ratio in 2011-2012 was 1.41 (95% CI:1.01, 1.95). Results are presented in Table 4.

## Discussion

This study evaluated the trends in frequency of sealant placement for children who lived in homes with someone who smoked inside the home from cross-sectional NHANES data from 2001-2002 and 2011-2012. The results indicate that, except for a significant increase in sealant placement in Non-Hispanic Blacks, there was no significant change in sealant placement for children who lived in homes with someone who smoked inside the home over the 10 years. In 2011- 12, 33.1% (SE=3.9) of children ages 6 to less than 12 years living in homes in which someone smoked inside the home received sealants; and31.0% (SE=4.8) of children ages 12 to less than 20 years living in homes in which someone smoked inside the home received sealants. When compared with children in whom no one smoked inside the home in logistic regression, children with someone who smoked inside the home were more likely to not have received sealants with both crude and adjusted odds ratios. The association remained as strong in the 2011-2012 analyses as it was in the 2001-2002 analyses even after controlling for sex, race/ethnicity, family income to poverty ratio, age, and insurance status.

This is the first study, to analyze the association of someone who smoked inside the home and sealant placement for the children who lived in the home. There have been significant cultural/behavioral changes in tobacco use and in smoking inside the home in the U.S. However, the remaining smokers are more likely to underutilize preventive dental care [19,21]. Bloom *et al*., reported that current smokers were twice as likely as former smokers/never smokers to not have had a dental visit in more than 5 years [21]. Iida *et al*., also reported that U.S. women of childbearing age who smoked were more likely to have untreated caries, an indicator of an individual's lack of preventive/routine care [22]. Similarly, Drilea *et al*., reported that 32.9% of current smokers had dental visits within the year as compared with 45.0% of non-smokers [23] and Mucci and Brooks reported lower dental services among long term cigarette smokers [24].

Additionally, Yeung *et al*., reported that overall, the use of health preventive services in children is not optimal, and was especially low for dental preventive services in young children [25]. Dye *et al*., suggested targeting self-care messages to smokers [19]. The results of this study additionally suggest that targeted messages to smokers should also include the importance of sealants as dental preventive services for children. Smoking in the home was an influential factor in children not having sealants placed and should be considered in public health discussions concerning dental care as well as in dentist/dental hygienist and patient communications.

This study has limitations. The determination of someone smoking in the home was a reported answer on a questionnaire. The report could be subject to social desirability bias. However, such a bias would be to respond that no one smoked inside the home and would tend to lower the association in this study. It would increase the likelihood that the null hypothesis would not be rejected. Also, the presence of someone smoking in the home does not necessarily indicate that the smoker is responsible for the healthcare of the child. Additionally, the variable for parental/guardian education level was not available. Parental/ guardian education could be a confounder with smoking inside the home and could be a limitation to the study. It should be noted that as a characteristic of cross-sectional study designs, causation and temporal relationships cannot be inferred.

The study also has strengths in that it used NHANES data which were collected from large and nationally representative samples by calibrated examiners in an oral health examination and by trained interviewers. The data are recognized as accurate and useful in producing epidemiological health statistics for the U.S. [20].

## Conclusion

Children with someone who smokes inside the home are less likely to have dental sealants than children who do not have someone who smokes inside the home. The relationship has remained unchanged from 2001-2002 to 2011-2012.

## Figures and Tables

**Table 1:** Sample Characteristics Children ages 6 to less than 20 years, NHANES 2001-2 and 2011-12

	2001-2			2011-12		
	N	Weighted Frequency	Weighted column % (SE)	N	Weighted Frequency	Weighted column % (SE)
Sample	3352			2374		
Sex	**p=.9842**					
Male	1691	27,706,116	**51.3** (0.9)	1214	28,492,615	**51.3** (1.4)
Female	1711	26,314,258	**48.7** (0.9)	1160	27,025,902	**48.7** (1.4)
Age groups in years	**p=.3293**					
6 less than 12	1152	23,709,978	**43.9** (1.2)	1204	23,365,136	**42.1** (1.4)
12 to less than 20	2250	30,310,396	**56.1** (1.2)	1170	32,153,382	**57.9** (1.4)
Race/ethnicity	**p=.6782**					
NHW	1011	32,359,376	**59.9** (3.0)	548	30,211,806	**54.4** (4.4)
NHB	1083	7,940,637	**14.7** (2.4)	705	8,377,572	**15.1** (3.0)
Mexican Am	1015	6,176,756	**11.4** (1.4)	441	7,978,456	**14.4** (2.8)
Other	293	7,543,604	**13.9** (2.8)	680	8,950,684	**16.0** (1.9)
Family income to poverty ratio	**p=.6748**					
0 to less than 1.25	1264	15,524,580	**29.8** (1.2)	996	17,694,202	**33.5** (3.8)
1.25 to less than 2.00	583	8,284,066	**15.9** (1.1)	364	8,254,779	**15.6** (1.7)
2.00 to less than 4.00	847	15,771,928	**30.3** (1.3)	475	14,373,295	**27.2** (2.8)
4.00 and above	554	12,498,461	**24.0** (1.5)	363	12,448,521	**23.6** (2.7)
Insurance	**p=.0841**					
Yes	2713	45,566,533	**84.5** (1.6)	2100	49,631,823	**89.6** (2.2)
No	678	8,371,126	**15.5** (1.6)	266	5,759,394	**10.4** (2.2)
Sealants, overall	**p=.0063**					
Yes	963	18,000,937	**33.1** (2.2)	879	22,876,797	**41.2** (1.9)
No	2439	36,019,436	**66.9** (2.2)	1495	32,641,721	**58.8** (1.9)
Sealants by age						
6 yrs to< 12 yrs	**p=.0085**					
yes	275	6,725,501	**28.4** (3.6)	431	9,228,363	**39.5** (1.9)
no	877	16,984,477	**71.6** (3.6)	773	14,136,774	**60.5** (1.9)
12 yrs to <20 yrs	**p=.1135**					
yes	688	11,275,436	**37.2** (1.9)	448	13,648,435	**42.4** (2.8)
no	1562	19,034,959	**62.8** (1.9)	722	18,504,947	**57.6** (2.8)
Smoking inside the home	**p<.0001**					
Yes	734	12,194,338	**22.6** (1.6)	294	6,059,806	**10.9** (1.1)
No	2668	41,826,035	**77.4** (1.6)	2080	49.458,711	**89.1** (1.1)

N=number; SE=standard error; NHW= Non-Hispanic White; NHB= Non-Hispanic Black; Am=American; and yrs-years. p-value is based on the Rao-Scott Chi-Square test between the years 2001-2 and 2011-12.

**Table 2:** Frequency of someone smoking inside the home and childhood experiences with sealants: Children ages 6 to less than 12 years, NHANES 2001-2 and 2011-12

	2001-2			2011-12			% difference in receiving sealants (2011-12)-(2001-2) and p-value
	N	Wt F	Wt column %^1^ (SE)	N	Wt F	Wt column %^2^ (SE)	
Overall, 6 to less than 12, in homes							
where someone smoked inside the home							
Yes, sealants	49	1,273,863	**21.8** (7.3)	56	984,330	**33.1** (3.9)	+11.3% **p=.2268**
No sealants	224	4,561,319	**78.2** (7.3)	108	1,985,621	**66.9** (3.9)	

Male							
Yes, sealants	26	733,168	**35.9** (9.2)	32	511,337	**31.3** (4.4)	- 4.6% **p=.6209**
No sealants	97	2,094,108	**74.1** (9.2)	55	1,124,476	**68.7** (4.4)	
Female							
Yes, sealants	23	540,695	**18.0** (6.1)	24	472,993	**35.5** (9.7)	+17.5% **p=.1356**
No sealants	127	2,467,211	**82.0** (6.1)	53	861,145	**64.5** (9.7)	

NHW							
Yes, sealants	16	817,224	**22.4** (8.1)	16	503,518	**31.0** (8.3)	+ 8.6% **p=.4642**
No sealants	74	2,833,184	**77.6** (8.1)	30	1,222,697	**69.0** (8.3)	
NHB							
Yes, sealants	21	204,365	**17.5** (4.3)	25	250,180	**31.4** (3.3)	+13.9% **p=.0080**
No sealants	103	965,621	**82.5** (4.2)	56	546,730	**68.6** (3.3)	
Mexican Am							
Yes, sealants	10	49,517	**13.9** (4.6)		*		
No sealants	62	307,576	**86.1** (4.6)				
Other race/ethnicity							
Yes, sealants	*			14	215,849	**59.1** (4.7)	
No sealants				12	149,566	**40.9** (4.7)	

0 to less than 1.25 family income to poverty ratio							
Yes, sealants	20	613,094	**21.0** (11.3)	39	628,865	**34.2** (7.8)	+13.2 **p=.4071**
No sealants	132	2,312,137	**79.0** (11.3)	74	1,210,576	**65.8** (7.8)	
1.25 to less than 2.00	*				*		
2.00 to less than 4.00							
Yes, sealants	15	384,087	33.3 (8.6)	10	272,355	**39.9** (2.2)	+6.6 **p=.4720**
No sealants	28	768,082	66.7 (8.6)	14	410,963	**60.1** (2.2)	
4.0 and above	*				*		

1 Weighted column percent for years 2001-2002;

2 Weighted column percent for years2011-2012.

* Results were suppressed due to cell size Abbreviations: N=number; Wt=weighted; F=frequency; SE=standard error; NHW= Non-Hispanic White; NHB= Non-Hispanic Black; Am=American.

p-value is based on the Rao-Scott Chi-Square test between the years 2001-2 and 2011-12.

**Table 3:** Someone smoking inside the home and childhood experience with sealants: Children ages 12 to less than 20 years NHANES 2001-2 and 2011-12

	2001-2			2011-12			% difference in receiving sealants (2011-12)-(2001-2) and p-value
	N	Wt F	Wt column %^1^ (SE)	N	Wt F	Wt column %^2^ (SE)	
Overall, 12 to less than 20, in homes							
where someone smoked inside the home							
Yes, sealants	177	1,889,362	**29.7** (2.5)	45	957,454	**31.0** (4.8)	+ 1.3% **p=.8141**
No sealants	344	4,469,794	**70.3** (2.5)	85	2,132,401	**69.0** (4.8)	

Male							
Yes, sealants	52	920,791	**28.8** (3.4)	34	712,143	**40.5** (5.9)	+11.7% **p=.0763**
No sealants	174	2,275,109	**71.2** (3.4)	36	1,047,426	**59.5** (5.9)	
Female							
Yes, sealants	65	968,571	**30.6** (3.9)	11	245,312	**18.4** (5.0)	-12.2% **p=.0644**
No sealants	170	2,194,686	**69.4** (3.9)	49	1,084,976	**81.6** (5.0)	

NHW							
Yes, sealants	59	1,479,449	**34.8** (3.3)	11	407,459	**24.2** (5.1)	-10.6% **p=.1192**
No sealants	98	2,771,774	**65.2** (3.3)	28	1,276,250	**75.8** (5.1)	
NHB							
Yes, sealants	42	254,721	**21.7** (6.0)	17	225,003	**28.6** (6.6)	+ 6.9% **p=.4409**
No sealants	161	920,997	**78.3** (6.0)	40	563,062	**71.4** (6.6)	
Mexican American							
Yes, sealants	10	49,517	**13.9** (4.6)		*		
No sealants	62	307.576	**86.1** (4.6)				
Other							
Yes, sealants		*		11	181,589	**44.0** (9.4)	
No sealants				15	230,652	**56.0** (9.4)	

0 to less than 1.25 family income to poverty ratio							
Yes, sealants	44	514,576	**20.3** (4.7)	30	569,846	**32.2** (4.5)	+11.9% **p=.0639**
No sealants	169	2,035,889	**79.7** (4.7)	56	1,197,584	**67.8** (4.5)	
1.25 to less than 2.00							
Yes, sealants	16	298,214	**26.3** (6.9)		*		
No sealants	65	835,270	**73.7** (6.9)				
2.00 to less than 4.00							
Yes, sealants	32	616,347	**40.8** (5.5)		*		
No sealants	62	892,862	**59.2** (5.5)				
4.00 and above							
Yes, sealants	16	318,819	**39.5** (6.7)		*		
No sealants	28	489,173	**60.5** (6.7)				

1 Weighted column percent for years 2001-2002;

2 Weighted column percent for years2011-2012.

* Results were suppressed due to cell size; Abbreviations: N=number; Wt=weighted; F=frequency; SE=standard error; NHW= Non-Hispanic White; NHB= Non-Hispanic Black; Am=American. p-value is based on the Rao-Scott Chi-Square test between the years 2001-2 and 2011-12.

**Table 4:** Logistic regression on not having a dental sealant with someone who smokes inside the home, NHANES 2001-2002 and 2011-12

	**Unadjusted Odds Ratios (95%CI)**	
	**2001-2002**	**2011-2012**
**Smoking in home**		
**Yes v. No**	**1.57 (1.17, 2.10)**	**1.56 (1.20, 2.03)**
	**Adjusted Odds Ratios (95% CI)**	
	**2001-2002**	**2011-2012**
**Smoking in home**		
**Yes v. No**	**1.31 (0.97, 1.78)**	**1.41 (1.01, 1.95)**
Family income to poverty		
0 to <1.25 v. 4.00 +	1.79 (1.22, 2.61)	1.21 (0.80, 1.83)
1.25 to <2.00 v. 4.00+	1.47 (1.08, 2.01)	1.04 (0.70, 1.55)
2.00 to <4.00 v. 4.00+	1.06 (0.72, 1.58)	1.38 (0.90, 2.12)
Race/ethnicity		
NHB v. NHW	1.85 (1.21, 2.81)	1.88 (1.38, 2.56)
Mexican Am v. NHW	1.50 (1.05, 2.14)	1.04 (0.74, 1.47)
Other v. NHW	1.85 (1.36, 2.51)	1.25 (0.91, 1.71)
Sex		
Male v. Female	1.00 (0.85, 1.19)	1.11 (0.83, 1.48)
Insurance		
Yes v. No	2.09 (1.41, 3.09)	1.22 (0.71, 2.11)
Age groups		
6 to < 12 v. 12 to <20	1.44 (1.04, 2.01)	1.15 (0.87, 1.53)

CI- confidence interval; v- versus; <- less than; +- and above; NHB- Non-Hispanic Black; NHW- Non-Hispanic White; Am- American.
